# ENAH‐202 promotes cancer progression in oral squamous cell carcinoma by regulating ZNF502/VIM axis

**DOI:** 10.1002/cam4.6652

**Published:** 2023-10-30

**Authors:** Xinyue Zhang, Xi Chen, Dongyuan Sun, Ning Song, Minmin Li, Wentian Zheng, Yang Yu, Gang Ding, Yingying Jiang

**Affiliations:** ^1^ School of Stomatology Weifang Medical University Weifang Shandong China; ^2^ Weifang Key Laboratory of Oral Biomedicine Weifang Medical University Weifang Shandong China

**Keywords:** cancer progression, ENAH‐202, epithelial‐to‐mesenchymal transition, long non‐coding RNA, oral squamous cell carcinoma, ZNF502

## Abstract

**Background:**

We aimed to demonstrate the regulatory effect of long non‐coding RNA (lncRNA) ENAH‐202 on oral squamous cell carcinoma (OSCC) development as well as its molecular mechanism.

**Methods:**

We detected ENAH‐202 expression in OSCC tissues and cell lines by quantitative real‐time PCR (qPCR). The biological function of ENAH‐202 was assessed in vitro and in vivo using CCK‐8, colony formation assays, transwell assays, xenograft formation, and tail vein injection. The further molecular mechanism by which ENAH‐202 promoted OSCC progression was identified using RNA pull‐down, LS‐MS/MS analysis, RNA immunoprecipitation (RIP), and chromatin immunoprecipitation (ChIP) assays.

**Results:**

ENAH‐202 was significantly upregulated in OSCC tissues and cells. ENAH‐202 promoted OSCC cell proliferation, migration, and invasion in vitro and in vivo. The expression of enabled homolog (ENAH) and epithelial‐to‐mesenchymal transition (EMT)‐related proteins was changed with the expression of ENAH‐202. Moreover, ENAH‐202 promoted the transcription of Vimentin (VIM) by binding with ZNF502, which can help ENAH‐202 promote OSCC progression.

**Conclusions:**

ENAH‐202 facilitated OSCC cell proliferation and metastasis by regulating ZNF502/VIM axis, which played an important role in OSCC progression.

## Graphical abstract

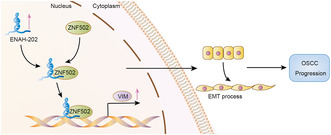

## 1 INTRODUCTION

The most common form of head and neck squamous cell carcinoma (HNSCC) is oral squamous cell carcinoma (OSCC).^1^ Globally, there are more than 300,000 new cases of OSCC each year, and more than 140,000 patients die from OSCC.^2^ Advanced OSCC shows high metastasis and recurrence rates and current traditional treatments cannot effectively improve the 5‐year survival rate.^3^ To enhance the prognosis of patients with OSCC, an early diagnosis is essential, and further exploration of the molecular mechanism to discover OSCC development to elevate the treatments is urgently needed.

Long non‐coding RNAs (lncRNAs) are a class of RNA molecules with a transcript length of more than 200 nucleotides that cannot encode proteins.^4^ The function of lncRNAs is related to lots of biological processes, for instance, histone modification, regulation of DNA methylation, and chromatin remodeling.^5^ It can be seen that lncRNAs are important for the occurrence and progression of cancers such as OSCC at transcriptional, post‐transcriptional, and translational levels.^6^ Therefore, lncRNAs may serve as biomarkers for the treatment and prognosis of OSCC patients because of their easy detectability and superior molecular stability.^7^ Therefore, it is urgent to further explore the expression and function of lncRNAs in OSCC, which can be used as the theoretical basis for prevention and therapy of OSCC patients.

In our previous study, we screened the differential lncRNA expression profiles of six OSCC and adjacent normal tissues using gene microarray and bioinformatics technology, and some lncRNAs that are highly expressed in OSCC were obtained (Figure S1).^8^ From the analysis of the results, we found that ENST00000358675 (also named ENAH‐202) was upregulated in OSCC tissues (Figure S1B). Through the query of Ensemble database with accession number ENST00000358675 (http://www.ensembl.org/Homo_sapiens/Transcript/Summary?db=core;g=ENSG00000154380;r=1:225495480‐225515397;t=ENST00000358675), it is found that ENAH‐202 is one of the transcripts of enabled homolog (ENAH), localized on human chromosome 1: 225,495,480‐225,515,397 reverse strand, and has nine exons.^9^ ENAH (also known as MENA or hMena), is a member of the enabled/vasodilator‐stimulated phosphoprotein (Ena/VASP) family,^10^ and has been proven to be highly expressed in various cancer types, such as breast cancer,^11^ gastric cancer,^12^ esophageal squamous cell carcinoma,^13^ ovarian cancer,^14^ etc. In the studies of OSCC, the high expression of ENAH in OSCC was associated with epithelial‐to‐mesenchymal transition (EMT) induced tumor progression and metastasis, as well as poor prognosis in OSCC patients.^15^, ^16^ The function and mechanism of ENAH‐202 in oral cancer are still unknown.

In this study, ENAH‐202 was shown to play a vital role in promoting OSCC cell proliferation, migration, and invasion in vitro and in vivo, and its underlying mechanism for influencing EMT was explored. This lays an experimental foundation for further exploring whether ENAH‐202 can act as a novel potential and predictive biomarker in patients with OSCC in the future.

## 2 MATERIALS AND METHODS

### 2.1 Specimens

In previous reports, the specimens and clinicopathological information of OSCC patients were described in detail, which were from “Sharing platform for the tissue sample and bioinformatics database of oral maxillofacial tumors” (Shanghai, China).^8^, ^17^

### 2.2 Cell lines and cell culture

In this study, the OSCC cell lines HN4, HN6, HN30, SCC‐4, SCC‐9, SCC‐25, and CAL‐27 were obtained as previously described.^18^ The cells grew under the standard conditions with atmosphere containing 5% CO_2_ at 37°C. HN4, HN6, HN30, and CAL‐27 were cultured in Dulbecco's modified Eagle's medium (DMEM; Gibco‐BRL, Grand Island, NY, USA). SCC‐4, SCC‐9 and SCC‐25 were cultured in DMEM/F12 (1:1) medium (Gibco‐BRL). In all media, 10% heat‐inactivated fetal bovine serum (FBS) (Gibco‐BRL), penicillin (100 units/mL), and streptomycin (100 μg/mL) (Invitrogen, Carlsbad, CA, USA) were added. Normal cells (human oral mucosa epithelial cells) were primary cultured in keratinocyte serum‐free medium (Gibco‐BRL) with 0.2 ng/mL recombinant epidermal growth factor (Invitrogen).^18^

### 2.3 RNA extraction and quantitative real‐time PCR (qPCR)

TRIzol reagent (Invitrogen) was used to extract total RNA following the protocol of the manufacturer. By using the PrimeScript RT kit (TaKaRa, Tokyo, Japan), total RNA was reverse transcribed into cDNA. We used an ABI 7500 Fast Real‐Time PCR System (Life Technologies, Carlsbad, CA, USA) and TB Green Premix Ex Taq reagent kits (Life Technologies) for qPCR. GAPDH was used as an internal control to calculate the relative RNA expression in each sample. Table S1 listed the sequences of the PCR primers we used in this study.

### 2.4 Western blotting

The cells were lysed and collected in tubes after 48 h transfection. The bicinchoninic acid assay (BCA) kits (Thermo Fisher Scientific, Waltham, MA, USA) were performed to determine protein concentrations. After electrophoresis, protein samples were transferred to PVDF membranes. Blocking the membranes with 5% non‐fat milk was then followed by immunoblotting with the primary antibodies against ENAH (1:1000, ab124685, Abcam, Cambridge, UK), E‐cadherin (1:500, ab15148, Abcam), GAPDH (1:1000, ab181602, Abcam), Vimentin (VIM) (1:1000, D21H3, CST, Danvers, MA, USA), and Flag Tag (1:1000, AF0036, Beyotime, Shanghai, China). Secondary antibodies were anti‐mouse and anti‐rabbit HRP‐labeled secondary antibodies (1:10000, CST). Visualization of the signals was carried out with ECLUltra (New Cell and Molecular Biotech, Suzhou, China).

### 2.5 Cell transfection and lentiviral transduction

The smart silencer/antisense oligonucleotides (ASOs) for ENAH‐202 knockdown were designed and synthesized by Guangzhou RiboBio Co., Ltd. (Guangzhou, China), siRNA for zinc finger protein (ZNF) 502 (si‐ZNF502) were designed by Designer of Small Interfering RNA (DSIR, http://biodev.extra.cea.fr/DSIR/DSIR.html)^19^ and synthesized by Sangon Biotech Co., Ltd. (Shanghai, China), and the sequences were showed in Table S2. ENAH‐202 lentiviral vector (LV‐ENAH‐202) and the ZNF502 plasmid with 3 × Flag‐tag (ZNF502‐Flag) were both constructed by HanYin Biotechnology Co., Ltd. (Shanghai, China). Cells transfection and lentiviral transduction were performed according to manufacturer's instructions as described.^20^

### 2.6 Cell Counting Kit‐8 (CCK‐8) assays

Cells transfected for 24 h or stably transduced with lentivirus vector were inoculated into 96‐well plate at the density of 1000 cells/well. Each group contained three replicate wells, and the cell viability was measured at a fixed time every day for 4 days. 10 μL cell Counting Kit‐8 (CCK‐8) reagents (Dojindo, Kuamoto, Japan) were added into the wells, incubated at 37°C for 2 h. Then the optical density (OD) was measured at 450 nm using a microplate reader (Shanpu, Shanghai, China).

### 2.7 Colony formation assays

Thousand cells were evenly inoculated in a six‐well plate and cultured at 37°C for 2 weeks. At room temperature, the colonies were fixed with 4% paraformaldehyde and dyed with 1% crystal violet solution. The colonies of more than 50 cells were counted under the microscope (Carl Zeiss, Oberkochen, Germany).

### 2.8 Cell migration and invasion assays

Twenty‐four‐well Transwell chambers (Corning, NY, USA) were used to measure cell migratory and invasive abilities that with a porosity polycarbonate filter (8 μm porosity) coated without or with the Matrigel (BD Biosciences, San Jose, CA, USA). After transfection 24 h, upper chamber of the transwell was filled with 150 μL of serum‐free cell suspension containing 5 × 10^4^ cells, while a lower chamber was filled with 600 μL of DMEM containing 10% FBS and placed on a well. Incubation at 37°C for 24–36 h was followed by fixation in 4% paraformaldehyde for 15 min and staining with 1% crystal violet for 30 min. Microscopic areas of five randomly selected stained cells per filter were captured under an invert phase‐contrast microscope (Carl Zeiss).

### 2.9 Isolation of nuclear and cytoplasmic fractionation

With the PARIS kit (Thermo Fisher Scientific), cellular, nuclear, and cytoplasmic RNA were isolated as directed by the manufacturer. The nuclear and cytoplasmic RNAs were purified, and the genomic DNA was removed by using DNase I (Beyotime). The nuclear and cytoplasmic RNA were reverse‐transcribed into cDNA and qPCR amplification was performed.^18^, ^21^ The endogenous control was U6 for the nucleus, and GAPDH for the cytoplasm.

### 2.10 Xenograft formation and in vivo metastasis assays

Weifang Medical University's Medical Laboratory Animal Center conducted all animal experiments adhere to the corresponding ethical standards and national guidelines. The animal studies were conducted on BALB/c nude 4‐week‐old mice.

To determine the effect of ENAH‐202 on tumor growth in vivo, 1 × 10^6^ HN6 cells stably transduced with LV‐ENAH‐202 and negative control (LV‐NC) were subcutaneously injected into the right and left flanks of six mice. The tumor growth was monitored every 3 days, and the observation was ended after 21 days when the largest tumor grew to about 10 mm. The mice were euthanized, the tumor was harvested and weighed. The paraffin‐embedded samples were stained with H&E and analyzed by immunohistochemistry (IHC). IHC staining of the sections was performed as previously described.^18^ Capture images using a light microscope (Olympus, Tokyo, Japan).

To assess whether ENAH‐202 knockdown could inhibit OSCC cell metastasis through tail vein to lung, the model of pulmonary metastasis via tail vein was carried out.^22^, ^23^ 1 × 10^6^ CAL‐27 cells were injected into the tail vein of each mouse (10 mice in total), and the in vivo ASO delivery was carried out every 4 days for six times. In brief, cholesterol‐conjugated ENAH‐202 ASO (ASO‐ENAH‐202) or negative control (ASO‐NC) were injected into two groups (five mice per group) via tail vein (10 nmol in 0.1 mL of saline buffer per mouse), respectively. After 7 weeks, the mice were euthanized and the lungs were harvested. Following that, we examined the lung metastatic nodules by picric acid and neutral aldehyde staining, as well as H&E staining.

### 2.11 RNA pull‐down assay and liquid chromatography tandem mass spectrometry (LC‐MS/MS)

RNA pull‐down assay was described in detail in previous studies.^18^, ^24^ Briefly, CAL‐27 cells stably transduced with LV‐ENAH‐202 were crosslinked with 1% formaldehyde and equilibrated with glycine buffer, then washed in cold PBS. The nuclear extract was isolated, lysed and sonicated. Then, the lysate was incubated with the ENAH‐202 biotin probe or negative probe as control (Guangzhou RiboBio Co., Ltd), and then incubated with streptavidin magnetic beads (Invitrogen). LC–MS/MS analysis was performed by OE Biotech (Shanghai, China) on precipitated proteins after washing and elution. After RNA pulldown assays, immunoblot analysis was used to confirm the proteins.

### 2.12 RNA immunoprecipitation‐qPCR assays

RNA immunoprecipitation assays (RIP) were performed using the RIP kit (Bersin Bio, Guangzhou, China). Briefly, cells were lysed by polysome lysis buffer, then DNA of the cells was removed. Flag or IgG antibody (Beyotime) was added into lysate and incubated overnight about 16 h at 4°C. Next day, protein A/G magnetic beads were added and incubated. RNA was extracted, purified, and dissolved. RNA was then reversed transcribed as described above, followed by qPCR and measured the enrichment of ENAH‐202.

### 2.13 Chromatin immunoprecipitation‐qPCR analysis

Chromatin immunoprecipitation (ChIP) assay was experimented using ChIP kit (Beyotime). Cells were crosslinked with 1% formaldehyde and equilibrated with glycine. Then, the SDS lysis buffer was used for lysis, and the DNA was sheared into 200–1000 bp by sonicator. The lysate was edulcorated, and then incubated with primary antibody (Flag or IgG) overnight at 4°C. Protein A/G agarose/salmon sperm was then added and incubated for 1 h next day. The beads were washed and eluted by elution buffer. Finally, DNA was purified by DNA purification kit (Beyotime) and analyzed by qPCR. The primers for ChIP‐qPCR were showed in Table S1.

### 2.14 Statistical analyses

For the analysis of all statistical data, SPSS version 16.0 (IBM, Armonk, NY, USA) and GraphPad Prism 7.0 (GraphPad Software, San Diego, CA, USA) were used. The measurement data were presented as a mean ± standard deviation (SD). We used one‐way analysis of variance (ANOVA) for comparisons among three or more groups and *t*‐tests for comparisons between two groups. The difference was considered statistically significant with a *p*‐value less than 0.05.

## 3 RESULTS

### 3.1 ENAH‐202 was highly expressed in OSCC and was mainly localized in nuclei

**FIGURE 1:**
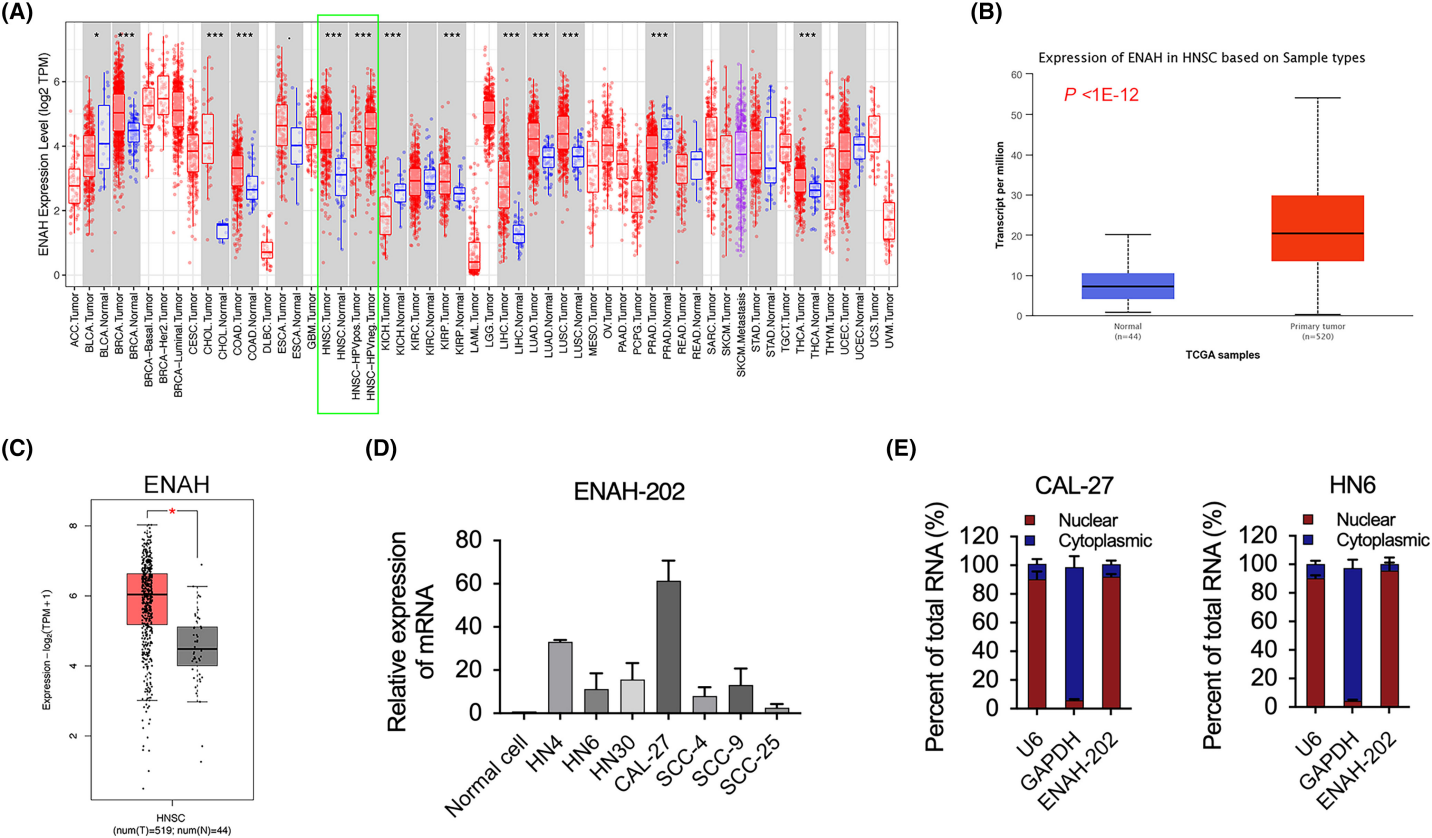
ENAH‐202 expression was upregulated in OSCC and was mainly localized in nuclei. (A) Expression of ENAH in multiple human cancers from TCGA determined in TIMER database. Green box shows the results of HNSC Dataset. (B) Expression of ENAH in HNSC from TCGA samples (44 normal vs. 520 primary tumor) shown in UALCAN database. (C) Expression of ENAH in HNSC from TCGA samples (44 normal vs. 519 primary tumor) shown in GEPIA2 database. (D) Relative expression of ENAH‐202 in seven OSCC cell lines was detected by qPCR compared to the normal oral epithelial cells (Normal Cell). (E) The distribution of ENAH‐202 in CAL‐27 and HN6 cells was measured by cell nucleus/cytoplasm fractionation. U6 and GAPDH served as endogenous controls. *p < 0.05.

By querying the expression of ENAH in pancancer through TIMER database (https://cistrome.shinyapps.io/timer/),^25^ it was found that ENAH was highly expressed in HNSC dataset (Figure 1A). The same results were also found in the UALCAN database (http://ualcan.path.uab.edu/analysis.html, Figure 1B)^26^ and the GEPIA2 database (http://gepia2.cancer‐pku.cn, Figure 1C). Furthermore, in seven OSCC cell lines, ENAH‐202 expression was higher than that in primary normal oral epithelial cells (normal cells) assessed by qPCR (Figure 1D). Cytoplasmic/nuclear fractionation was used to verify the subcellular localization of ENAH‐202 in OSCC cells, and ENAH‐202 was primarily located in the nucleus according to the results (Figure 1E). According to these results, ENAH‐202 expression was increased in OSCC and localized in nuclei of OSCC cells.

### 3.2 Knockdown of ENAH‐202 suppressed cell proliferation and metastasis of OSCC cells in vitro

**FIGURE 2:**
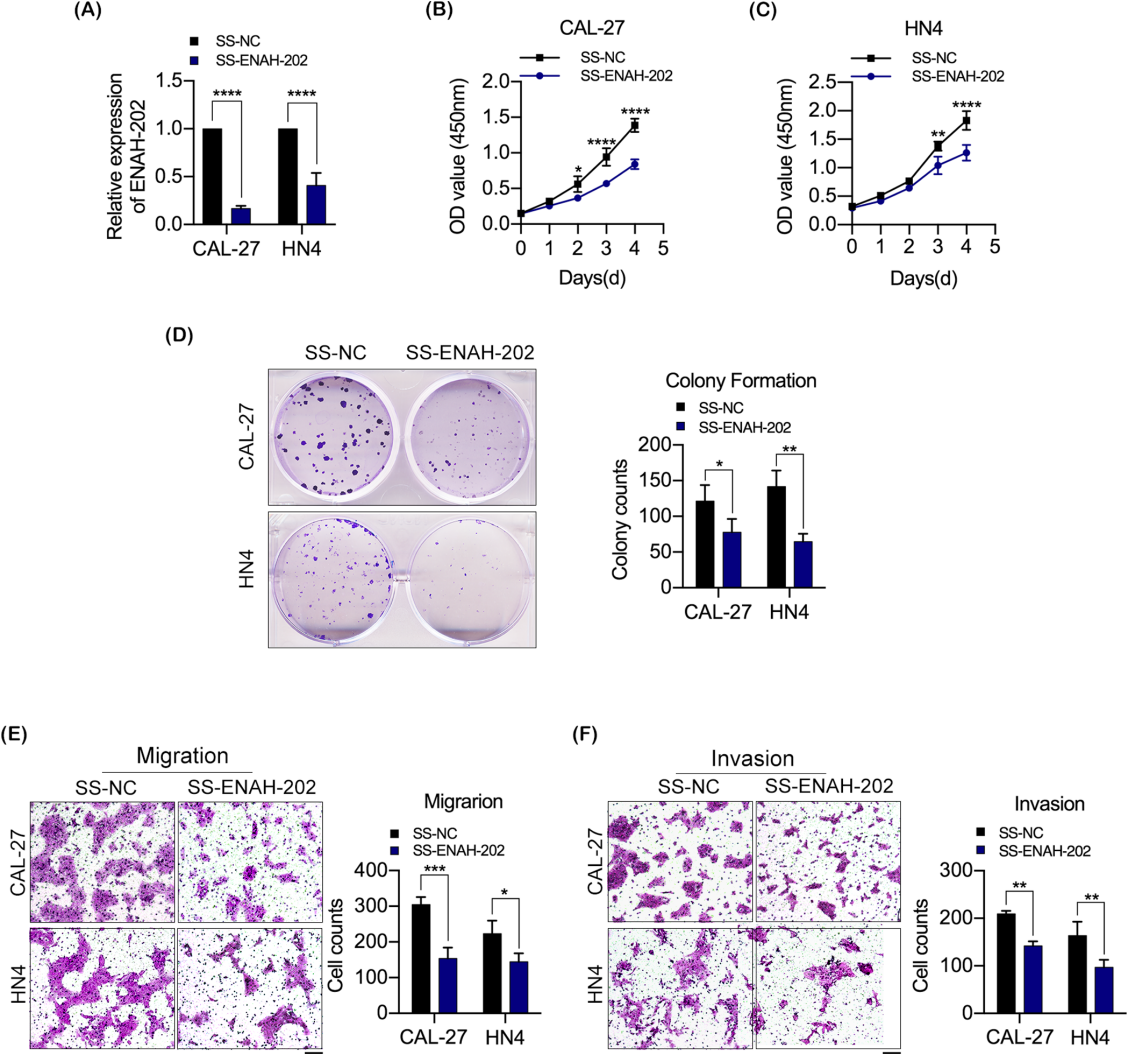
ENAH‐202 knockdown inhibited cell proliferation and metastasis of OSCC cells in vitro. (A) The knockdown efficiency was analyzed by qPCR in CAL‐27 and HN4 cells transfected with ENAH‐202 smart silencer (SS‐ENAH‐202). (B–D) Effects of ENAH‐202 on cell proliferation were detected by CCK‐8 (B, C) and colony formation assays (D) in CAL‐27 and HN4 cells transfected with SS‐ENAH‐202. (E, F) Transwell assays were used to measure the migration (E) and invasion (F) abilities of CAL‐27 and HN4 cells transfected with SS‐ENAH‐202. Scale bar: 100 μm. *p < 0.05, **p < 0.01, ***p < 0.001, ****p < 0.0001.

To determine the effects of ENAH‐202 knockdown on cell proliferation and metastasis, the knockdown efficiency of ENAH‐202 smart silencer (SS‐ENAH‐202) should be clarified first. The relative expression of ENAH‐202 in CAL‐27 and HN4 cells transfected with SS‐ENAH‐202 at 24 h was determined by qPCR (Figure 2A). As determined by CCK‐8 and colony formation assays, ENAH‐202 knockdown resulted in inhibition of OSCC cell proliferation (Figure 2B–D). Moreover, ENAH‐202 knockdown significantly reduced OSCC cell migration and invasion as determined by transwell assays (Figure 2E,F).

### 3.3 ENAH‐202 overexpression promoted proliferation and metastasis of OSCC cells in vitro

**FIGURE 3:**
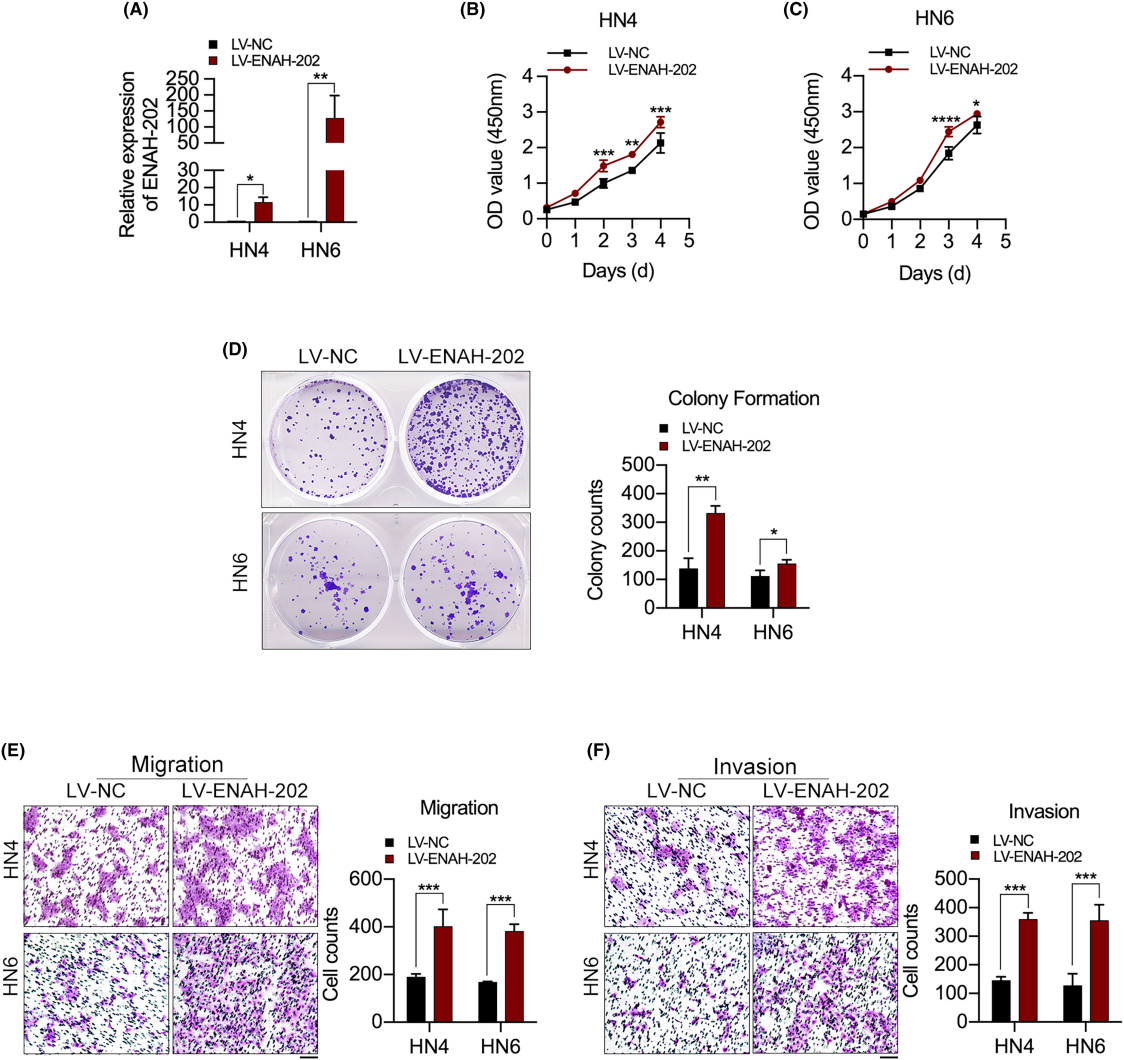
ENAH‐202 overexpression facilitated cell proliferation, migration and invasion of OSCC cells in vitro. (A) The relative expression of ENAH‐202 in HN4 and HN6 cells stably transduced with the ENAH‐202 lentiviral vector (LV‐ENAH‐202) were measured by qPCR. (B–D) Effects of ENAH‐202 on cell proliferation were detected by CCK‐8 (B, C) and colony formation assays (D) in HN4 and HN6 cells stably transduced with LV‐ENAH‐202. E, F. Transwell assays were used to measure the migration (E) and invasion (F) abilities of HN4 and HN6 cells stably transduced with LV‐ENAH‐202. Scale bar: 100 μm. *p < 0.05, **p < 0.01, ***p < 0.001, ****p < 0.0001.

HN4 and HN6 cells were stably transduced with ENAH‐202 lentiviral vectors (LV‐ENAH‐202) and ENAH‐202 overexpression efficiency was confirmed by qPCR (Figure 3A). As measured by CCK‐8 and colony formation assays, the cell growth was greatly enhanced after ENAH‐202 overexpression (Figure 3B‐D). Moreover, ENAH‐202 overexpression facilitated the migratory and invasive abilities of OSCC cells as determined by Transwell assays (Figure 3E, F).

### 3.4 ENAH‐202 promoted cell growth and metastasis of OSCC in vivo

**FIGURE 4:**
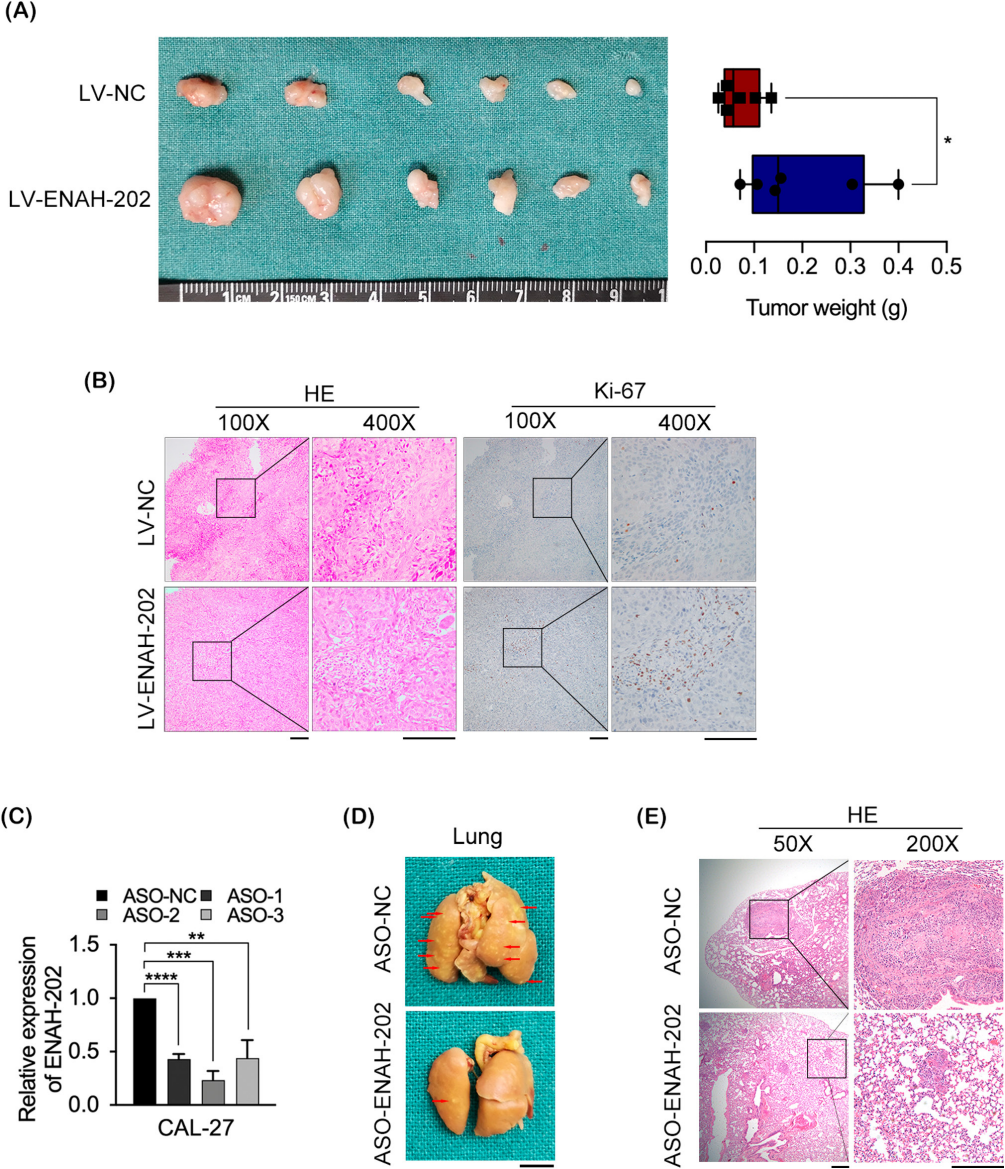
ENAH‐202 promoted cell growth and metastasis of OSCC in vivo. (A) Tumor volume and weight of ENAH‐202 overexpression were measured in nude mice subcutaneously injected with HN6 cells. *n* = 6/group. (B) H&E and Ki‐67 staining of xenograft tissues. Scale bar: 50 μm. (C) The silence efficiency of ASO‐ENAH‐202 in CAL‐27 cells was determined by qPCR. (D) Typical images of the lungs of mice injected with CAL‐27 cells stably transduced with ASO‐ENAH‐202 or ASO‐NC via tail vein for 7 weeks. The red arrows show yellow nodules on the lung surfaces. Scale bar: 5 mm. (E) Representative images of H&E staining of lung tumor tissues. Scale bar: 100 μm. *p < 0.05, **p < 0.01, ***p < 0.001, ****p < 0.0001.

In comparison with the LV‐NC group, subcutaneous injection of HN6 cells stably transduced with LV‐ENAH‐202 into nude mice had significantly larger tumor volumes and weights (Figure 4A). Further verification of these results was provided by H&E and Ki‐67 staining (Figure 4B). The mice were sacrificed 8 weeks after injecting tail veins, and the metastatic nodules on their lungs were examined. ENAH‐202 silence efficiency of three ASOs in CAL‐27 cells was measured by qPCR, ASO‐2 (ASO‐ENAH‐202) was used for the follow‐up in vivo ASO delivery (Figure 4C). The results indicated that the number of tumor nodules in ASO‐ENAH‐202 group on the lung surfaces was less than that in the control group (Figure 4D). Furthermore, H&E staining showed that the size of metastatic nodules was reduced in mice bearing ENAH‐202‐silenced cells (Figure 4E).

### 3.5 ENAH‐202 was positively correlated with ENAH expression and promoted the process of EMT

**FIGURE 5:**
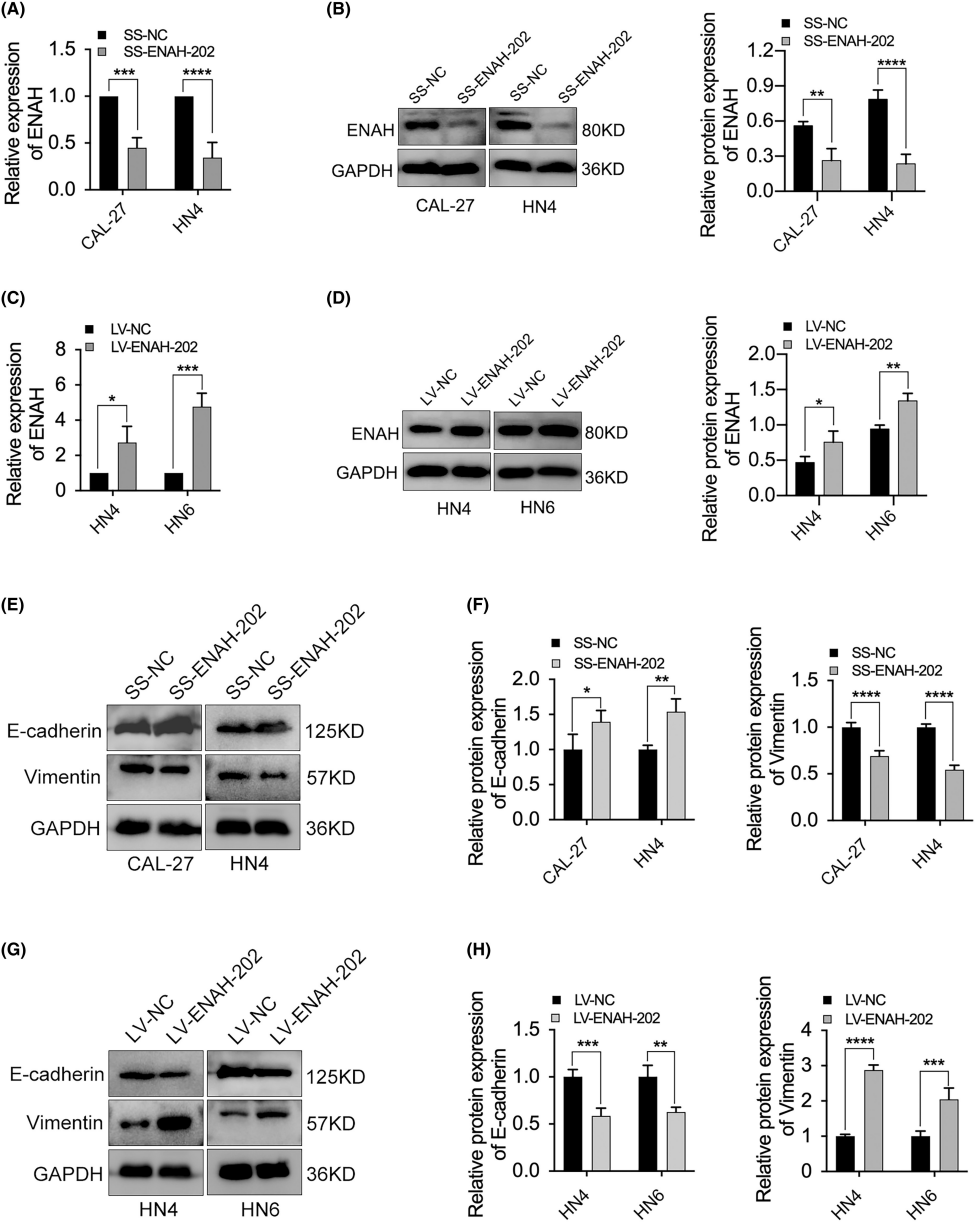
Effect of ENAH‐202 on the expression of ENAH and the process of EMT in OSCC cells. (A, B) Effect of ENAH‐202 knockdown on the mRNA and protein expression of ENAH in CAL‐27 and HN4 cells by qPCR and western blotting. (C, D) Effect of ENAH‐202 overexpression on the mRNA and protein expression of ENAH in HN4 and HN6 cells by qPCR and western blotting. (E, F) Effect of ENAH‐202 knockdown on the protein expression of E‐cadherin and Vimentin. (G, H) Effect of ENAH‐202 overexpression on the protein expression of E‐cadherin and Vimentin. *p < 0.05, **p < 0.01, ***p < 0.001, ****p < 0.0001.

When ENAH‐202 was knocked down in CAL‐27 and HN4 cells, the mRNA and protein expression of ENAH were both decreased (Figure 5A,B). In contrary, the mRNA and protein expression of ENAH were obviously elevated after ENAH‐202 overexpression in HN4 and HN6 cells (Figure 5C,D). It is thus clear that ENAH‐202 expression was positively correlated with ENAH.

To evaluate the effect of ENAH‐202 on the progress of EMT, E‐cadherin and Vimentin were determined by western blotting. The results showed that ENAH‐202 knockdown in CAL‐27 and HN4 cells could suppress the protein expression of Vimentin and increase the protein expression of E‐cadherin (Figure 5E,F). When ENAH‐202 was overexpressed in HN4 and HN6 cells, the protein expression of E‐cadherin was significantly decreased, and the level of Vimentin was significantly increased (Figure 5G,H).

### 3.6 ENAH‐202 accelerated the progress of OSCC by regulating ZNF502/VIM axis

**FIGURE 6:**
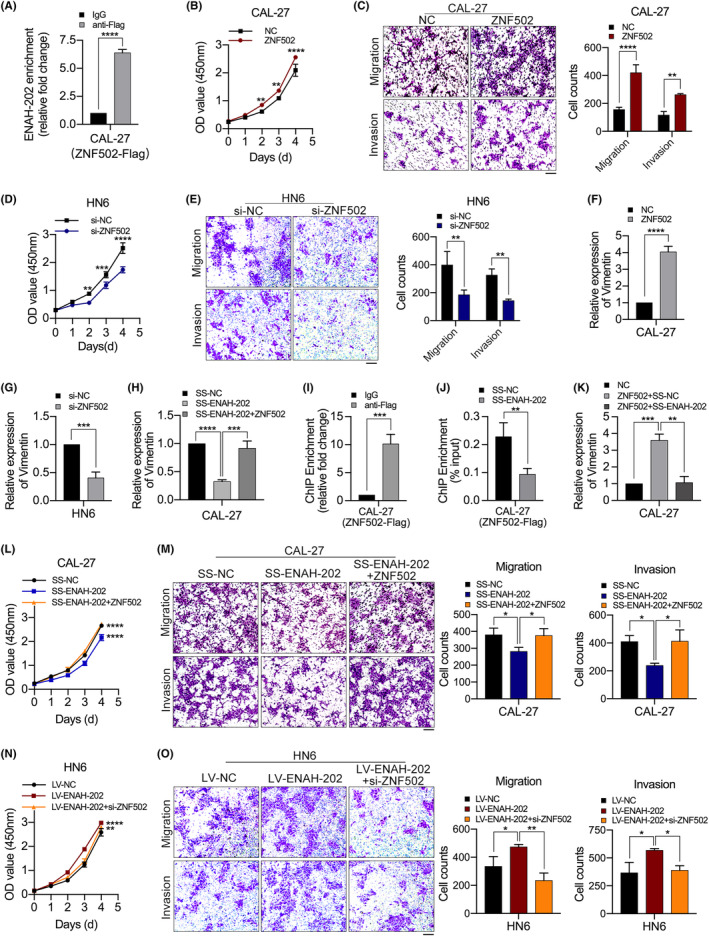
ENAH‐202 affected the progress of OSCC by regulating ZNF502/VIM axis. (A) qPCR analysis of ENAH202 enriched with ZNF502 in CAL‐27 cells transfected with ZNF502‐Flag vector in RIP assays. (B) Cell proliferation in CAL‐27 cells transfected with ZNF502‐Flag vector was measured using CCK‐8 assays. (C) Migratory and invasive ability in CAL‐27 cells transfected with ZNF502‐Flag vector were assessed using Transwell assays. (D) Cell proliferation in HN6 cells transfected with si‐ZNF502 was measured using CCK‐8 assays. (E) Migratory and invasive ability in HN6 cells transfected with si‐ZNF502 were assessed using Transwell assays. (F) The relative expression of Vimentin (VIM) in CAL‐27 cells transfected with ZNF502‐Flag vector was detected by qPCR. (G) The relative expression of VIM in HN6 cells transfected with si‐ZNF502 was detected by qPCR. (H) The relative expression of VIM was measured in ZNF502‐overexpression CAL‐27 cells transfected with SS‐ENAH‐202. (I) ChIP‐qPCR analysis of ZNF502 genomic occupancy of the VIM promoter in CAL‐27 cells transfected with ZNF502‐Flag. (J) ChIP‐qPCR analysis of ZNF502 genomic occupancy of the VIM promoter in CAL‐27 (ZNF502‐Flag) cells transfected with SS‐ENAH‐202. (K) qPCR analysis of the expression of VIM in CAL‐27 (ZNF502‐Flag) cells transfected with SS‐ENAH‐202. (L) Cell proliferation determined by CCK‐8 assays in ZNF502‐overexpression CAL‐27 cells transfected with SS‐ENAH‐202. (M) Cell migration and invasion detected by using Transwell assays in ZNF502‐overexpression CAL‐27 cells transfected with SS‐ENAH‐202. (N) Cell proliferation determined by CCK‐8 assays in ZNF502‐knockdown HN6 cells transduced with LV‐ENAH‐202. (O) Cell migration and invasion detected by using Transwell assays in ZNF502‐knockdown HN6 cells transduced with LV‐ENAH‐202. Scale bar: 100 μm. *p < 0.05, **p < 0.01, ***p < 0.001, ****p < 0.0001.

**FIGURE 7:**
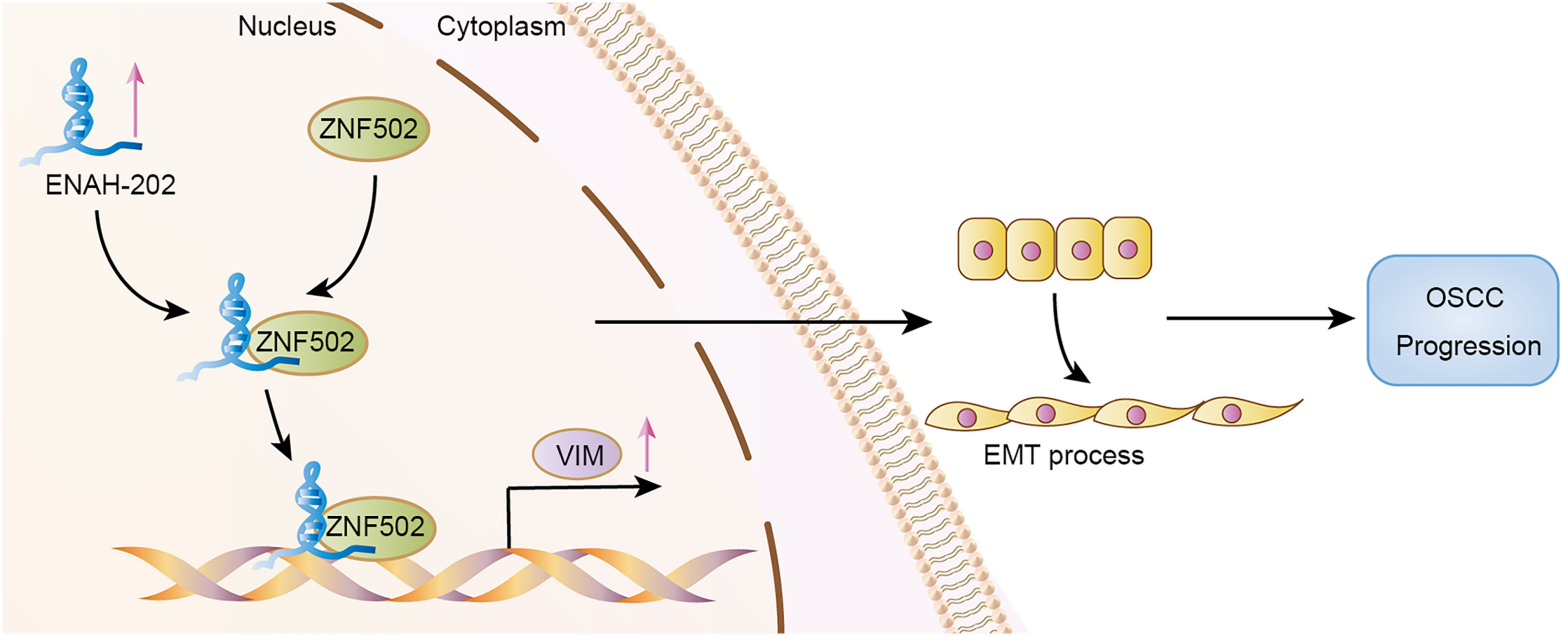
Proposed model shows the regulatory effect of ENAH‐202 in OSCC progression.

In order to explore the interaction between ENAH‐202 and its RNA‐binding proteins (RBPs), RNA pull‐down assays and mass spectrometry were performed (https://proteomecentral.proteomexchange.org/cgi/GetDataset?ID=PXD040379), and the peptide sequence of ZNF502 protein was identified by secondary mass spectrometry (Figure S2A). Bioinformatics analysis via RNA‐Protein Interaction Prediction website (RPISeq; http://pridb.gdcb.iastate.edu/RPISeq/)^27^ revealed that ZNF502 might serve as a binding protein of ENAH‐202 (Figure S2B). ZNF502‐Flag plasmid and si‐ZNF502 was constructed for subsequent experiment. The overexpression efficiency of ZNF502‐Flag plasmid (Figure S2C,D) and the knockdown efficiency of si‐ZNF502 (Figure S2E) were verified. RIP experiment was used to determine whether ZNF502 could bind to ENAH‐202, and the results determined a significant enrichment of ENAH‐202 with ZNF502 (Figure 6A). To evaluate the function of ZNF502 on OSCC progression, we determined the cell proliferation and metastasis using CCK‐8 assays and transwell assays in ZNF502‐overexpression CAL‐27 cells and ZNF502‐knockdown HN6 cells. The results showed that cell proliferation, migration, and invasion were boosted when ZNF502 was overexpressed (Figure 6B,C) and suppressed when ZNF502 was knocked down (Figure 6D,E). In addition, as the expression of ZNF502 increased, the mRNA level of Vimentin (VIM) also elevated (Figure 6F), and vice versa (Figure 6G). ZNF502 overexpression reversed the expression of VIM in SS‐ENAH‐202 CAL‐27 cells (Figure 6H). In order to further explore the role of ZNF502 as a transcription factor, the binding motif was queried by JASPAR 2022 database (https://jaspar.genereg.net/),^28^ and the binding sites for ZNF502 in the VIM promoter were predicted (Figure S2F). To verify the binding of ZNF502 to the VIM promoter, ChIP assays were performed. The ChIP assay results showed that ZNF502 enrichment in the VIM promoter region significantly increased (Figure 6I), and ENAH‐202 knockdown could suppress the binding of ZNF502 to the VIM promoter (Figure 6J). Meanwhile, ENAH‐202 knockdown could inhibit the increased expression of VIM caused by ZNF502 overexpression (Figure 6K). In addition, ZNF502 overexpression could restore OSCC cell proliferation, migration and invasion ability in SS‐ENAH‐202 CAL‐27 cells (Figure 6L,M), and similarly, ZNF502 knockdown could restore OSCC cell proliferation, migration and invasion ability in LV‐ENAH‐202 HN6 cells (Figure 6N,O). The above results suggest that the interaction of ENAH‐202 and ZNF502 can enhance the transcription of VIM to affect the EMT process, thus accelerating the progress of OSCC (Figure 7).

## 4 DISCUSSION

In recent years, the incidence of OSCC was gradually increasing and associated with various parameters including gender, lifestyle, and living region.^29^ Conventional diagnosis and treatment for patients with OSCC are still unfavorable.^30^ The importance of early diagnosis and treatment of OSCC patients is becoming increasingly recognized in order to improve their long‐term survival rate and prognosis.^31^ Previous studies demonstrated that lncRNAs acting as novel biomarkers participated in numerous biological processes and were related to the progress of OSCC.^18^, ^22^, ^32^ However, the specific molecular mechanism underlying OSCC progression requires further investigation.

In this study, elevated expression of lncRNA ENAH‐202 was confirmed in OSCC tissues and cell lines. Furthermore, loss‐ and gain‐of‐function experiments indicated that ENAH‐202 could enhance the cell proliferation and metastasis of OSCC in vitro and in vivo, which might provide an important theoretical basis for screening and identifying potential biomarkers for the diagnosis and treatment of OSCC.

As a transcript of ENAH gene, ENAH‐202 can affect the expression of host gene ENAH, which suggests that ENAH‐202 may have the same effect as ENAH. According to the researches of ENAH in a variety of cancer types, ENAH could promote cancers progression and was correlated with the occurrence of EMT.^15^, ^33^, ^34^, ^35^ In this study, ENAH‐202 had obvious effect on OSCC cell invasion and migration. EMT process has been emphasized as a reversible dynamic process, which plays a critical role during tumor development and metastasis.^36^ Epithelial cells gain mesenchymal phenotypes as they lose their apical‐basal polarity and cell–cell contacts, which is associated with increased ability to migrate and invade.^37^, ^38^ In addition, previous studies demonstrated that lncRNAs were involved in the promotion of OSCC occurrence and development via EMT process.^18^, ^21^, ^39^, ^40^ In this study, the effect of ENAH‐202 on the protein expression of E‐cadherin (epithelial markers) and Vimentin (mesenchymal markers) were investigated, and the results confirmed that ENAH‐202 promoted EMT process.

It has been shown that lncRNAs are capable of regulating gene expression by interacting with DNA, RNA, or proteins.^41^ Our previous research found that the aberrantly expressed lncRNA in OSCC could play its role in promoting cancer by combining with its RNA‐binding protein. It found that lnc‐POP1‐1 promotes OSCC cells cisplatin resistance by interacting with MCM5.^24^ Also, LINC00460 could bind to PRDX1 and promote EMT‐related genes expression to regulate OSCC progression.^18^ In this study, ZNF502 was revealed to bind to ENAH‐202 based on the results of mass spectrometry analysis and database validation, and the combination was further verified by RIP assays. ZNF502 is a member of the zinc finger protein family, the largest family of DNA‐binding proteins in the human genome and encoded by 2% of human genes,^42^ which plays an important role as transcriptional activators or inhibitors.^43^ It is found that ZNF502 is related to acute myeloid leukemia,^44^ depression,^45^ and respiratory syncytial virus replication.^46^ However, ZNF502 is still rarely seen in cancer research. In this study, the function of ZNF502 in OSCC was explored. It was found that ZNF502 significantly enhanced OSCC cell proliferation and metastasis, which was consistent with the function of ENAH‐202. It suggested that the ENAH‐202 affected the progression of OSCC by interacting with ZNF502. ENAH‐202 could promote the EMT process, which was caused by ZNF502's regulation of VIM transcription, and ENAH‐202 could affect the regulation of VIM by ZNF502. ENAH‐202 could promote the EMT process, which was caused by the regulation of VIM transcription by ZNF502. Notably, ENAH‐202 could affect the regulation of VIM transcription by ZNF502. Therefore, it was preliminarily confirmed that ENAH‐202 could promote the EMT process by regulating ZNF502/VIM axis, thus affecting the progress of OSCC. However, further research is needed to clarify the development mechanism of OSCC.

## 5 CONCLUSIONS

ENAH‐202 was upregulated in OSCC and promoted cell proliferation and metastasis in vitro and in vivo. ENAH‐202 facilitated the progress of EMT by regulating ZNF502/VIM axis. This study provides an experimental basis for screening and identifying biomarkers for diagnosis and treatment of OSCC.

## AUTHOR CONTRIBUTIONS

**Xinyue Zhang:** Investigation (equal); methodology (equal); resources (equal); writing – original draft (equal). **Xi Chen:** Investigation (equal); methodology (equal); writing – original draft (equal). **Dongyuan Sun:** Funding acquisition (supporting); writing – original draft (equal); writing – review and editing (supporting). **Ning Song:** Investigation (equal); resources (equal); software (supporting). **Minmin Li:** Data curation (supporting); resources (supporting); software (supporting). **Wentian Zheng:** Resources (supporting); visualization (supporting). **Yang Yu:** Data curation (supporting); resources (supporting). **Gang Ding:** Funding acquisition (equal); project administration (equal); writing – review and editing (supporting). **Yingying Jiang:** Funding acquisition (equal); project administration (equal); writing – original draft (supporting); writing – review and editing (lead).

## FUNDING INFORMATION

This study was supported by grants from the National Natural Science Foundation of China (82103008), Shandong Provincial Natural Science Foundation (ZR2023LSW019, ZR2020MH192, ZR2022QH122, ZR2021MH051). This study was also supported by 2021 Youth Innovation Talent Introduction and Education Program of Shandong Province Universities (for YYJ) and the National Facility for Translational Medicine (Shanghai) (TMSF‐2021‐2‐003).

## CONFLICT OF INTEREST STATEMENT

The authors have no conflicts of interest to declare.

## ETHICS STATEMENT

The Ethics Committee of Weifang Medical University approved our study. Written informed consent was provided by the participants prior to enrollment. All experimental methods abided by the Helsinki Declaration.

## Supporting information


Figure S1.

Table S1.

## Data Availability

Data supporting the findings of this study are available within the paper and its Supplementary files. The mass spectrometry proteomics data have been deposited to the ProteomeXchange Consortium (http://proteomecentral.proteomexchange.org)^47^, ^48^ via the iProX partner repository with the dataset identifier PXD040379.
