# Supplementary material for: ENAH‐202 promotes cancer progression in oral squamous cell carcinoma by regulating ZNF502/VIM axis
Source: Cancer Med. 2023 Oct 30;12(22):20892–905. doi: 10.1002/cam4.6652 (PMC10709750; doi:10.1002/cam4.6652)
Supplement: Supplementary file 1 — Figure S1. [file CAM4-12-20892-s002.docx]

**Supplementary Figures**

**
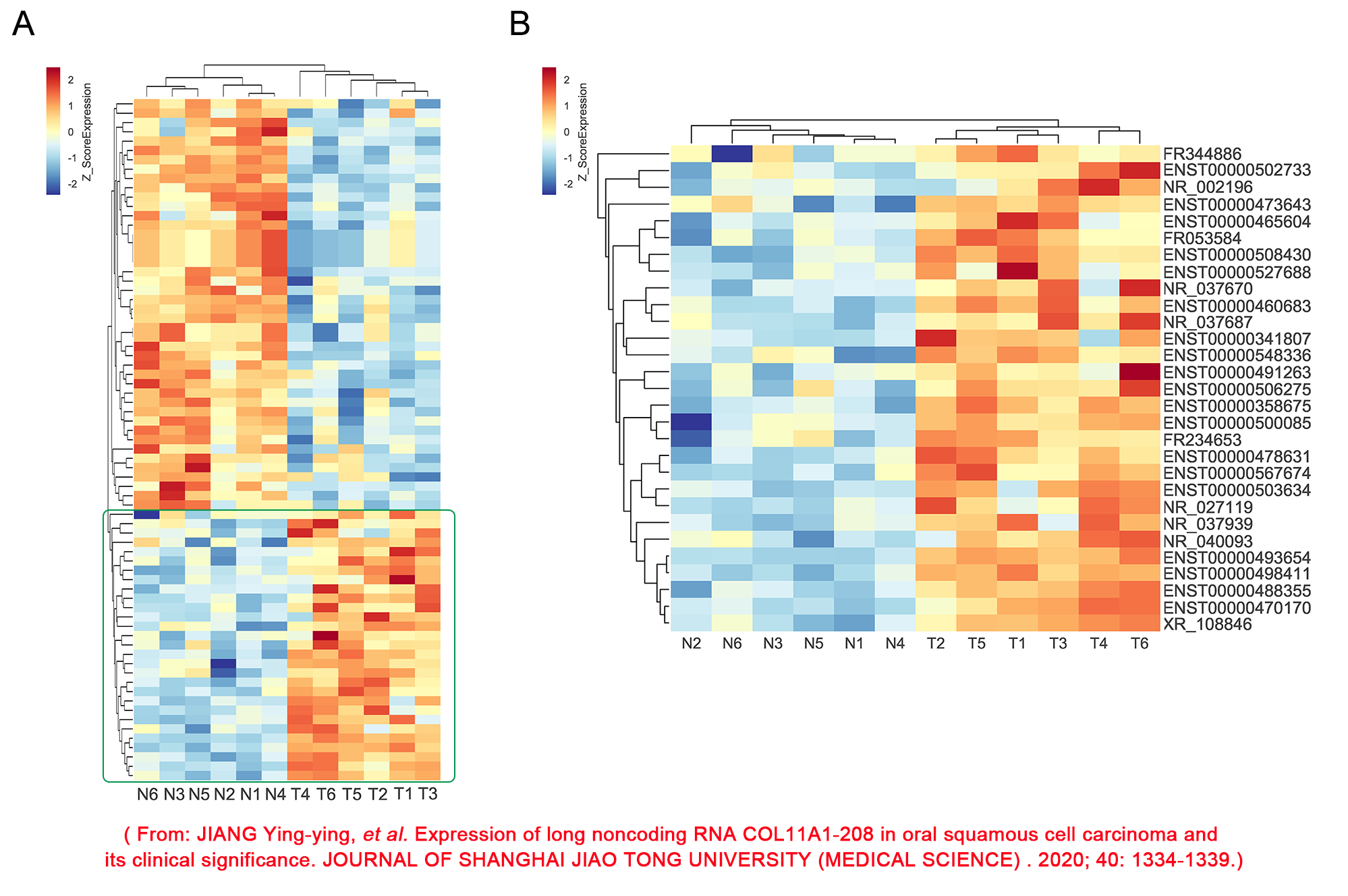
**

**Figure S1.** LncRNAs differentially expressed in six pairs of OSCC tissues and adjacent normal tissues.

A. Microarray analysis to assess the lncRNA gene expression profiles of six pairs of OSCC tissues (T) and adjacent normal tissues (N). The green frame shows the upregulated lncRNAs in OSCC tissues. B. Microarray analysis of the 30 upregulated lncRNAs in OSCC tissues.


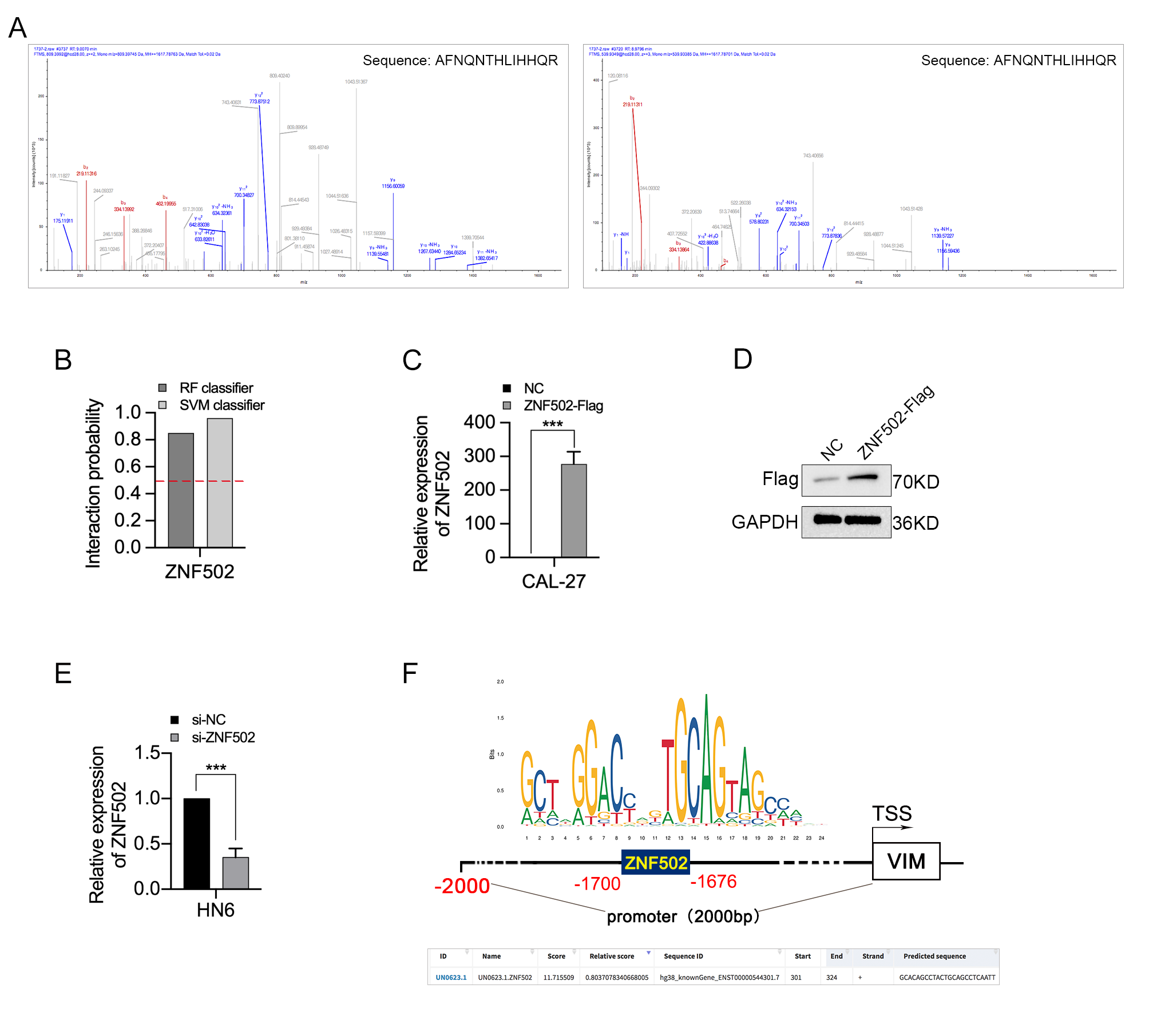


**Figure S2.** The transcription factor ZNF502 may bind to ENAH-202 and VIM promoter.

1. Following RNA pull-down assays, Secondary mass spectrum of Peptide sequence for ZNF502 were showed. B. RPIseq analysis was used to predicted the interaction probability between ENAH-202 and ZNF502 (RF and SVM classifier predictions with probabilities > 0.5 were considered positive interaction). C. The overexpression efficiency of ZNF502-Flag vector in CAL-27 cells was detected by qPCR. D. The overexpression efficiency of ZNF502-Flag vector in CAL-27 cells was detected by western blotting. E. The knockdown efficiency of si-ZNF502 in HN6 cells was detected by qPCR. F. The ZNF502 binding motif and predicted binding sites in the VIM promoter was determined with JASPAR database. ***p<0.001.
