# Supplementary material for: ENAH‐202 promotes cancer progression in oral squamous cell carcinoma by regulating ZNF502/VIM axis
Source: Cancer Med. 2023 Oct 30;12(22):20892–905. doi: 10.1002/cam4.6652 (PMC10709750; doi:10.1002/cam4.6652)
Supplement: Supplementary file 2 — Table S1. [file CAM4-12-20892-s001.docx]

**Supplementary Tables**

**Table S1.** Sequences of qPCR primers.

| **Primers** | | **Sequences (5'-3')** |
| --- | --- | --- |
| ENAH-202 | Forward | GGACCTTACATTTCAGTATCTTTCA |
|  | Reverse | TGCCTGAGAGAGAGAAATGTTAAGT |
| ENAH | Forward | TCTATCACCATACAGGCAACAAC |
|  | Reverse | GCACAGTTTATCACGACCTGA |
| GAPDH | Forward | GAACGGGAAGCTCACTGG |
|  | Reverse | GCCTGCTTCACCACCTTCT |
| U6 | Forward | CTCGCTTCGGCAGCACATATACT |
|  | Reverse | ATTTGCGTGTCATCCTTGCGCA |
| E-cadherin | Forward | CGAGAGCTACACGTTCACGG |
|  | Reverse | GGGTGTCGAGGGAAAAATAGG |
| Vimentin | Forward | AGTCCACTGAGTACCGGAGAC |
|  | Reverse | CATTTCACGCATCTGGCGTTC |
| ZNF502 | Forward | AGGAAGGAGGTTTTGGGAGAA |
|  | Reverse | AGTGAGATGTGAGTGATTGCG |
| VIM for ChIP-qPCR | Forward | GAGACAGGTTCTCACTCTGTTGCAC |
|  | Reverse | TTGCTTGAGCCTGGGAAATTGAGG |

**Table S2.** Sequences of Smart Silencer, ASO and siRNA.

| **Product Name** | **Sequences** |
| --- | --- |
| ENAH-202 Smart Silencer | CTAACTGCCATCCTATATAC |
|  | CTTGGACCTTACATTTCAGT |
|  | TCTGCGTACCTTCCCACATA |
|  | CGCTTACCTCTCTTCCAAA |
|  | CTAAAGCTCTAACCTAAAG |
|  | GGACCTTACATTTCAGTAT |
| ASO-ENAH-202-1 | CTAACTGCCATCCTATATAC |
| ASO-ENAH-202-2 ^*^ | CTTGGACCTTACATTTCAGT |
| ASO-ENAH-202-3 | TCTGCGTACCTTCCCACATA |
| si-ZNF502 | CCAAGATTCTACATTTGAAGA |

^*^ ASO for cholesterol-conjugated ASO-ENAH-202 for *in vivo* ASO delivery.
